# Overexpression of *GhGSTF9* Enhances Salt Stress Tolerance in Transgenic *Arabidopsis*

**DOI:** 10.3390/genes15060695

**Published:** 2024-05-27

**Authors:** Huimin Li, Yihui Liu, Jie Wu, Kexin Chang, Guangqiang Zhang, Hang Zhao, Nianwei Qiu, Ying Bao

**Affiliations:** 1 Suzhou Polytechnic Institute of Agriculture, Suzhou 215008, China; hmli0621@163.com; 2 College of Life Sciences, Qufu Normal University, Qufu 273165, China; 15610292665@163.com (Y.L.); 15254800887@163.com (K.C.); hangzhaolgl@163.com (H.Z.); nianweiqiu@163.com (N.Q.); 3 Cash Crop Research Institute of Jiangxi Province, Jiujiang 332105, China; wjie19910319@126.com; 4 College of Agriculture and Bioengineering, Heze University, Heze 274015, China; zhangbotanyphd@163.com

**Keywords:** cotton, salt stress, glutathione S-transferase, *GhGSTF9*

## Abstract

Soil salinization is a major abiotic stress factor that negatively impacts plant growth, development, and crop yield, severely limiting agricultural production and economic development. Cotton, a key cash crop, is commonly cultivated as a pioneer crop in regions with saline-alkali soil due to its relatively strong tolerance to salt. This characteristic renders it a valuable subject for investigating the molecular mechanisms underlying plant salt tolerance and for identifying genes that confer salt tolerance. In this study, focus was placed on examining a salt-tolerant variety, E991, and a salt-sensitive variety, ZM24. A combined analysis of transcriptomic data from these cotton varieties led to the identification of potential salt stress-responsive genes within the glutathione S-transferase (GST) family. These versatile enzyme proteins, prevalent in animals, plants, and microorganisms, were demonstrated to be involved in various abiotic stress responses. Our findings indicate that suppressing *GhGSTF9* in cotton led to a notably salt-sensitive phenotype, whereas heterologous overexpression in Arabidopsis plants decreases the accumulation of reactive oxygen species under salt stress, thereby enhancing salt stress tolerance. This suggests that *GhGSTF9* serves as a positive regulator in cotton’s response to salt stress. These results offer new target genes for developing salt-tolerant cotton varieties.

## 1. Introduction

Soil salinization, a significant environmental factor impeding plant growth and productivity, is a primary contributor to decreased crop yields [1,2]. Currently, global saline-alkali land covers 954 million hectares, constituting 10% of the world’s landmass. Excessive soil salt induces osmotic stress, ion toxicity, oxidative stress, and other forms of plant damage, disrupting vital physiological processes [3,4]. Given the increasing pressure on arable land, the development of salt-tolerant crop varieties and the enhancement of crop salt tolerance on saline-alkali land have become critical objectives.

In recent years, considerable progress has been made in understanding the mechanisms of plant salt tolerance [5,6]. Studies elucidate how plants integrate external salt signals and internal developmental cues to regulate growth, development, and tolerance to salt stress. This regulatory mechanism involves multiple transcription factor families, including MYB, WRKY, and NAM/ATAF/CUC (NAC) [7,8,9], along with phytohormone, such as abscisic acid (ABA), auxin (AUX), brassinosteroid (BR), and gibberellin (GA) [10,11,12,13]. Notably, different transcription factors exhibit varying responses to salt stress. For instance, R2R3-type MYB transcription factors predominantly participate in ABA- or MAPK-mediated salt stress tolerance, while AtMYB73 in *Arabidopsis thaliana* dampens plant salt tolerance by inhibiting the salt overly sensitive (SOS) pathway [14,15]. WRKY transcription factors modulate the salt response of the SOS pathway by regulating the expression of SOS pathway genes [16].

Besides transcription factors, plants also bolster their resistance to ROS damage induced by salt stress by elevating the activity of antioxidant enzymes [17]. Research demonstrates that glutathione transferase (GST), a superfamily comprising multiple functional proteins, can eliminate reactive oxygen species and contribute to plant resilience against various abiotic stresses [18,19]. In *Arabidopsis*, AtGSTU19 boosts salt stress tolerance by enhancing antioxidant enzyme activity and increasing proline content [20]. In alfalfa, GsGSTU13 mitigates oxidative damage caused by salt stress on cell membranes and sustains plant growth in adverse conditions [21]. In cotton, analysis of GST family gene expression patterns indicates that salt stress significantly triggers or inhibits the expression of GST genes, highlighting the diverse roles of GST family genes in cotton’s response to salt stress mechanisms [22,23]. However, the extent of the involvement of GST family genes in salt stress tolerance in cotton remains unclear.

Cotton, being a significant cash crop [24], exhibits noticeable salt tolerance traits. Hence, it serves as a valuable material for investigating the molecular mechanisms underlying plant salt tolerance and isolating salt tolerance genes. These genes can offer insights into plants’ strategies for adapting to salt stress [25,26,27]. With the rapid advancement of cotton genome research, several functional genes involved in the response to salt stress have been identified [8]. Studies have shown that overexpressing the transcription factors GhDof1 and GhABF2 in cotton enhances its tolerance to salt stress [28,29]. Overexpression of GhCIPK6a increases the expression level of co-expressed genes induced by salt stress [30]. Additionally, the ectopic overexpression of the GaJAZ1 gene in upland cotton significantly boosts the salt tolerance of transgenic lines [31]. Nevertheless, the known resources of salt tolerance genes in cotton remain limited. Thus, extensively exploring salt tolerance-related genes in cotton and employing genetic engineering methods to breed salt-tolerant varieties have emerged as effective strategies to combat salt stress and enhance the utilization efficiency of saline-alkali lands.

In this study, by analyzing the transcriptomes of the salt-tolerant variety E991 and the salt-sensitive variety ZM24, we identified numerous GST family genes potentially responsive to salt stress. Knocking down GSTF9 in cotton decreased salt stress tolerance while overexpressing *GSTF9* in *Arabidopsis* notably improved salt stress tolerance. These findings suggest that GSTF9 could play a significant role as a positive regulatory factor in enhancing salt stress tolerance in cotton, offering valuable genetic resources for the development of salt-tolerant varieties.

## 2. Materials and Methods

### 2.1. Plant Material and Salt Treatments

For salt treatment experiments of cotton material, silenced cotton plants and ZM24 plants were subjected to salt stress treatment using 200mM NaCl and cultured in the substrate at 28 °C (light)/23 °C (dark) with a long-day (LD, 16 h light/8 h dark) photoperiod for 15 days, respectively. For salt treatment experiments in Arabidopsis, WT and overexpression Arabidopsis plants were cultivated on a solid medium with different NaCl concentrations (0 mM, 150 mM, and 175 mM) for 20 days and 27 days, and then observed the root and leaf phenotypes, respectively.

### 2.2. RNA Extraction and RT-qPCR

The total RNA was extracted using a plant RNA extraction kit (DP441, Tiangen, Beijing, China) following the manufacturer’s instructions. The RNA concentration and purity of each sample were quantified using a NanoDrop ND-1000 spectrophotometer (NanoDrop, Wilmington, DE, USA). The first-strand cDNA was synthesized using HiScript II Q RT SuperMix (+gDNA wiper, R223-01;Vazyme, Nanjing, Jiangsu, China). with the manufacturer’s instructions. RT-qPCR assays were further conducted using MonAmp SYBR Green qPCR Mix Kit (Monad Biotech, Wuhan, China) in a LightCycler 96 PCR machine (Roche, Beijing, China). Gene expression values were calculated as described previously [32].

### 2.3. RNA-Seq and Data Analysis

The leaves from the same position of 28-day-old seedlings (ZM24 and E991) were carefully harvested for RNA-seq assays. Three biological replicates were collected for RNA-seq analysis. The Illumina Novaseq™ 6000 system was used for RNA-seq at LC-Bio (Hangzhou, China). The fragments per kilobase of transcript per million mapped reads (FPKM) method was used to determine the value of gene expression levels. DESeq2 was employed for differentially expressed genes (DEGs) analyses with fold-change (FC) > 2 and a value of false discovery rate (FDR) < 0.05.

### 2.4. Plasmid Construction and Transformation of Cotton

For virus-induced gene silencing (VIGS), the fragment of GhGSTF9 was synthesized (Sangon, Shanghai, China) and then ligated into BamHI- and SacI-digested the plant expression vector pCLCrVB to produce the *GhGSTF9*-silenced vector. The vectors were transformed into Agrobacterium strain GV3101 and then infiltrated into the cotyledons of 10-day-old cotton seedlings. After infiltration for 2 d in dark conditions, the plants were grown in a controlled environment at 28 °C (light)/23 °C (dark) under LD (16 h light/8 h dark) photoperiod conditions.

### 2.5. Arabidopsis Transformation

The GhGSTF9 coding sequences were retrieved from CottonGen. Genes were amplified from the ZM24 cDNA library and inserted into a expression vector driven by the CaMV 35S promoter. The vector was subsequently transformed into *Arabidopsis* Col-0 wild-type plants via the *Agrobacterium*-mediated floral-dip method.

### 2.6. Statistical Analysis

All quantitative analyses were repeated at least three times, and the data were expressed as the mean ± SD unless noted otherwise. Statistical significance was determined using Student’s *t*-test. * *p* < 0.05 was considered significant, and ** *p* < 0.01 were considered extremely significant.

## 3. Result

### 3.1. Identification of Genes Associated with Salt Stress Response in Cotton

**Figure 1:**
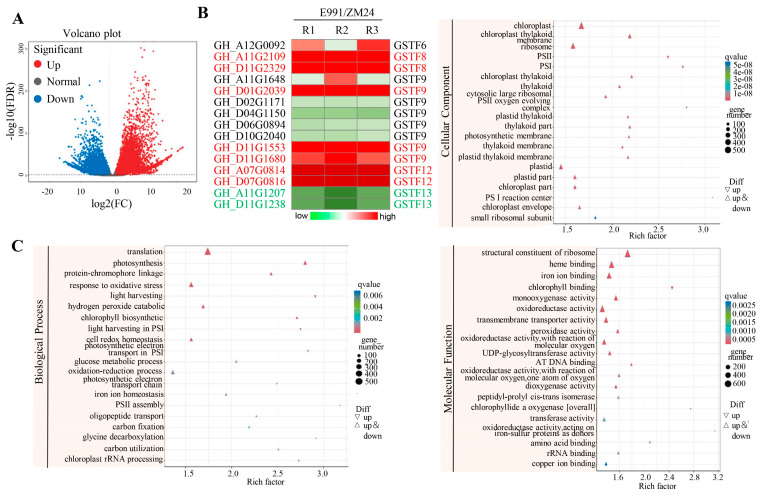
**Transcriptomic profiles of salt-sensitive and salt-tolerant cotton varieties.** (**A**) The DEGs in salt-tolerant cotton varieties E991 compared with salt-sensitive cotton varieties ZM24. (**B**) Heatmap showing the expression profiles of selected GST family genes in E991 plants compared with ZM24 plants. R1, R2, and R3 represent three replicates of RNA-seq assays. (**C**) GO analysis of DEGs between E991 and ZM24.

To investigate potential regulatory genes in cotton in response to salt stress, RNA-seq analysis was conducted on leaves from 4-week-old salt-tolerant cotton variety E991 and salt-sensitive variety ZM24, both grown under long-day (LD) conditions. A total of 23,141 differentially expressed genes (DEGs) were detected in the E991/ZM24 comparison, comprising 11,852 up-regulated and 11,289 down-regulated genes with fold-change (FC) > 2 and false discovery rate (FDR) < 0.05 (Figure 1A). Notably, the expression of several GST family genes, including GSTF8, GSTF9, GSTF12, and GSTF13, showed significant alterations in E991 compared to ZM24 plants (Figure 1B).

**Figure 2:**
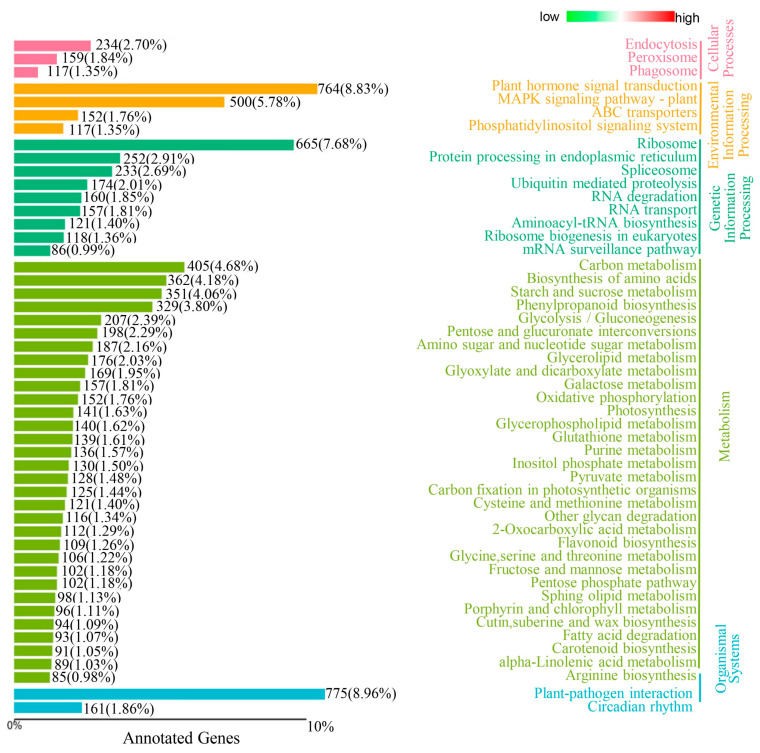
KEGG enrichment analysis of the DEGs in E991 compared with ZM24 plants.

A GO analysis was conducted to elucidate the functions of the DEGs). The analysis revealed enrichment in several crucial processes, including photosynthesis, translation, oxidation-reduction, and response to oxidative stress (Figure 1C). These pathways likely play a role in the response to salt stress to some extent. Additionally, a KEGG enrichment analysis was performed to further investigate the functions of the DEGs (Figure 2). The analysis showed enrichment of differential metabolites in E991/ZM24 across various pathways, such as amino acid metabolism, plant hormone signal transduction, carbohydrate metabolism, and oxidative phosphorylation. These findings suggest that the salt-tolerant cotton variety E991 may respond to salt stress by modulating the metabolism of several crucial pathways compared to the salt-sensitive cotton variety ZM24.

### 3.2. Silencing GhGSTF9 Reduces Salt Stress Resistance in Cotton

**Figure 3:**
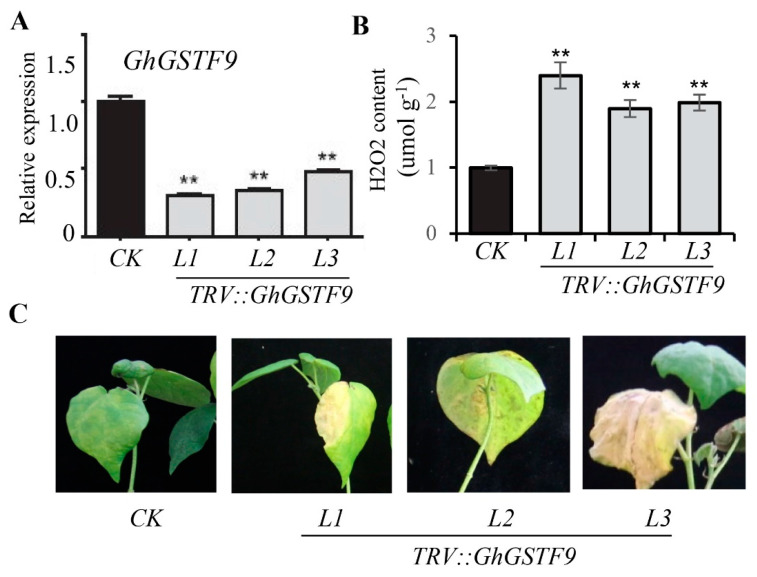
**Silencing of GhGSTF9 in cotton considerably weakened resistance to salt stress.** (**A**) RT−qPCR detection of relative expression of *GhGSTF9* in *GhGSTF9* silenced plants and ZM24 plants. Values are mean ± SD (n = 3). ** *p* < 0.01. (**B**) The concentration of H2O2 in GhGSTF9 silenced plants and ZM24 plants after 200mM salt stress. Values are mean ± SD (n = 5). ** *p* < 0.01. (**C**) Leaf morphology of *GhGSTF9* silenced plants and ZM24 plants after 200 mM salt stress.

Considering the significant differential expression of numerous GhGST family genes in the E991/ZM24 comparison, it is hypothesized that this gene family may play a role in the response of cotton to salt stress. To investigate the involvement of GhGST in the salt stress response, the VIGS method was employed to reduce the expression of *GhGSTF9* (GH_D11G1680) in the ZM24 variety (Figure 3). Fourteen days after Agrobacterium injection, the positive control GbCLA1 plants exhibited an albino phenotype, indicating the suitability of VIGS plants for subsequent experimental analyses. RT-qPCR experiments further validated the silencing of *GhGSTF9* (Figure 3A). Subsequently, the aforementioned materials were subjected to salt stress treatment using 200mM NaCl for 15 days. Phenotypic analysis revealed that *GhGSTF9*-silenced plants exhibited significant defoliation, wilting, and leaf yellowing symptoms compared to control materials (Figure 3B). Additionally, the silenced plants demonstrated higher H_2_O_2_ content relative to the control plants (Figure 3C). These findings suggest that silencing the *GhGSTF9* gene in cotton increases the sensitivity of cotton to salt stress.

### 3.3. Overexpression of GhGSTF9 Increases the Salt Stress Resistance in Arabidopsis

**Figure 4:**
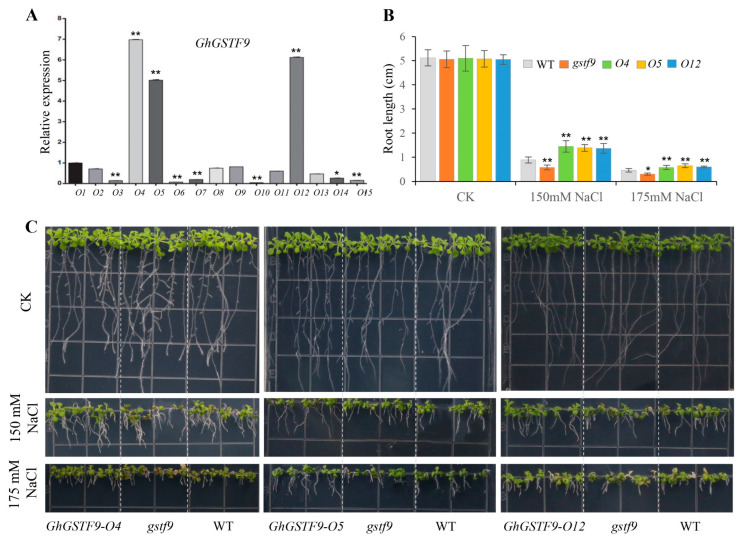
**Root length of Arabidopsis transgenic overexpressing *GhGSTF9* under salt stress.** (**A**) RT-qPCR detection of relative expression of GhGSTF9 in WT and *GhGSTF9-OE* transgenic lines. Values are mean ± SD (n = 3). * *p* < 0.05; ** *p* < 0.01. (**B**) The root length statistics of *GhGSTF9* transgenic Arabidopsis lines and Col-0 Arabidopsis on MS plates supplemented with 0 mM, 150 mM, and 175 mM NaCl. Values are mean ± SD (n = 5). * *p* < 0.05; ** *p* < 0.01. (**C**) The phenotypes of Arabidopsis with 0m M, 150 mM, 175 mM NaCl.

To further examine the role of *GhGSTF9* in responding to salt stress, we produced Arabidopsis lines overexpressing *GhGSTF9* to observe their traits and analyze their physiology. We assessed salt tolerance in three homozygous transgenic Arabidopsis lines from the T3 generation (lines 1, 6, and 14) with elevated *GhGSTF9* expression (Figure 4A). These transgenic lines were cultivated on a solid medium with different NaCl concentrations (0 mM, 150 mM, and 175 mM). Under normal conditions, there was no significant difference in root length between WT and transgenic plants. However, when exposed to 150 mM and 175 mM NaCl in MS medium, the root length of the three *GhGSTF9*-overexpressing lines (*GhGSTF9*-4, *GhGSTF9*-5, and *GhGSTF9*-12) was notably longer compared to the Col-0 Arabidopsis line (Figure 4B,C).

**Figure 5:**
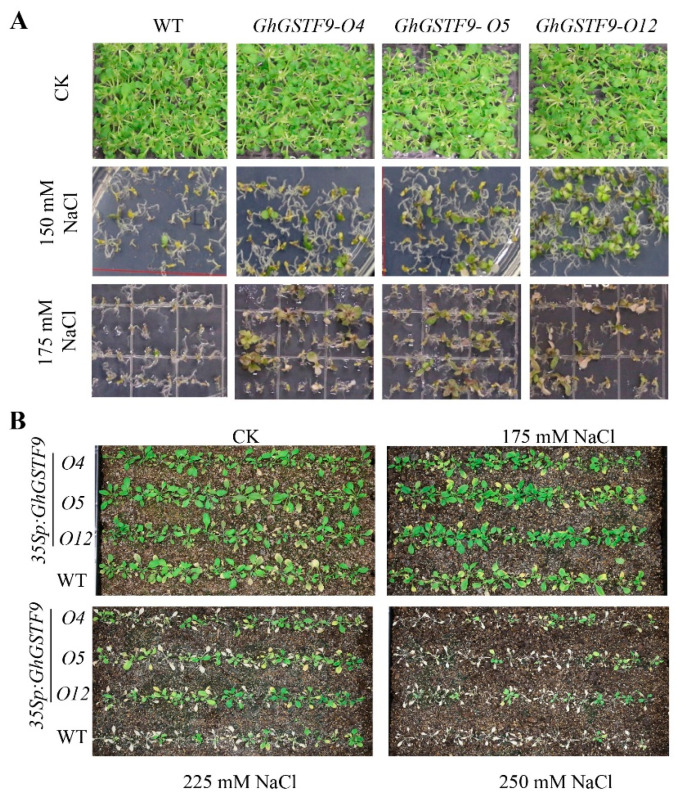
**Overexpression of the *GhGSTF9* gene increases salt tolerance in transgenic *Arabidopsis*.** (**A**) Seed germination of *GhGSTF9* transgenic *Arabidopsis* lines and Col-0 Arabidopsis on MS plates supplemented with 0 mM, 150 mM, and 175 mM NaCl. (**B**) Phenotype of GhGSTF9 transgenic *Arabidopsis* lines and WT under salt stress.

The germination of the *GhGSTF9*-overexpressing lines and WT plants on MS solid medium supplemented with 0 mM, 150 mM, or 175 mM NaCl was also compared. Without salt, no difference in seed germination was observed between the WT and GhGSTF9-overexpressing lines, with all the seeds germinating (Figure 5A). However, in MS medium supplemented with 150 mM and 175 mM NaCl, the percentage of germinated seeds from the three GhGSTF9-overexpressing lines exceeded that of WT plants, revealing a significant disparity between Col-0 and *GhGSTF9*-overexpressing transgenic lines (Figure 5A). These findings suggest that overexpressing *GhGSTF9* enhances the salt stress tolerance of transgenic Arabidopsis.

In a shared pot, both *GhGSTF9*-overexpressing transgenic lines and WT Arabidopsis plants underwent cessation of watering for one week following four weeks of regular growth. The soil received irrigation with water solutions containing 0 mM, 175 mM, 225 mM, and 250 mM NaCl, respectively, until the soil was fully saturated, after which the salt solution was drained. Subsequent observations revealed that leaves of WT Arabidopsis nearly desiccated entirely after exposure to 225 mM and 250 mM NaCl for 10 days, while a majority of leaves from the transgenic *Arabidopsis* remained verdant (Figure 5B). These findings provide further evidence supporting GhGSTF9’s potential role as a positive regulator of salt stress tolerance.

## 4. Discussion

Soil salinization presents significant environmental challenges that threaten agricultural production globally [33]. Cotton, an important cash crop in China, is often planted as a pioneer crop in saline-alkali regions due to its relatively high salt tolerance. However, the salt tolerance of cotton is limited, and its yield suffers under salt stress. In this study, the gene *GhGSTF9* was identified as potentially responsive to salt stress (Figure 1 and Figure 2). The silencing of this gene in cotton resulted in a significantly salt-sensitive phenotype (Figure 3), whereas its heterologous overexpression in Arabidopsis plants markedly improved salt stress tolerance, suggesting that *GhGSTF9* functions as a positive regulator in cotton’s response to salt stress (Figure 4 and Figure 5).

### 4.1. Involvement of the GST Gene Family in Cotton’s Response to Salt Stress

When plants encounter salt stress, oxidative metabolism within the plants escalates, resulting in the production of an abundance of ROS, which can inflict damage on the plant. GSTs are a class of multifunctional enzyme proteins that are found extensively in animals, plants, and microorganisms. These enzymes can function as part of the antioxidant enzyme clearance system, scavenging ROS and thus providing protection against various biotic and abiotic stresses [18,34]. Previous research has highlighted the significant role of the GST gene family in conferring resistance to salt stress in various plants such as rice, tomato, alfalfa, and soybean. For example, the heterologous transformation of the rice *OsGSTU4* gene into Arabidopsis increased the GST activity in transgenic plants, decreased ROS accumulation, and consequently enhanced salt stress tolerance [35]. The transformation of the tomato *LeGSTU2* gene into Arabidopsis significantly improved the salt tolerance of transgenic Arabidopsis plants [36], while *Glycine soja* *GsGSTU14*, *GsGSTU19*, and *GsGSTU13* positively influenced the salt and alkali stress tolerance of transgenic alfalfa [21]. Although the involvement of the GST gene family in plant salt stress tolerance has been documented in multiple species, the functional roles in cotton’s response to salt stress are not well understood. In this study, through the transcriptomic analysis of the salt-tolerant cotton variety E991 and the salt-sensitive variety ZM24, differential expression of many GST family genes was observed, indicating their potential as regulatory factors in cotton’s response to salt stress.

Interestingly, some of these differentially expressed genes, such as *GhGST8*, *GhGST9*, and *GhGST12*, exhibited high expression levels in the salt-tolerant varieties, while others, such as *GhGST13*, were highly expressed in the salt-sensitive varieties (Figure 1). This finding suggested that genes within the GhGST family might play both positive and negative regulatory roles in cotton’s response to salt stress. This observation aligns with previous findings that GST family genes can function as either positive or negative regulators in stress response pathways. For example, previous research demonstrated that overexpression of *PeGSTU8* in Arabidopsis plants could enhance plant tolerance to adversity by activating the antioxidant system [37], whereas the *CsGSTU45* gene negatively influenced tea plants’ resistance to pathogens [38]. In this study, it was confirmed that *GhGSTF9* significantly improved transgenic plants’ tolerance to salt stress (Figure 3, Figure 4 and Figure 5). Therefore, continuing the identification of GhGST family genes that contribute to salt stress tolerance is crucial for the selection of salt-tolerant cotton varieties in the future.

### 4.2. The GST Gene Family Exhibits Strong Potential for Application in Polyploid Breeding of Crops

In recent years, transgenic technology has made significant strides in developing single-trait crops, imbuing them with desirable qualities such as salt tolerance, insect resistance, drought resistance, and herbicide resistance. Despite these advances, field crops often face multiple abiotic stresses simultaneously, highlighting the need to cultivate superior crop varieties capable of withstanding diverse abiotic stresses together. Yet, the current approach to crop breeding frequently focuses on enhancing one desirable trait, which might compromise another due to the negative correlation between many desirable traits and the limitations of target genes utilized in molecular breeding [39,40]. Consequently, achieving the synergistic optimization of multiple desirable traits to produce high-quality, multi-resistant crop varieties poses a considerable challenge in crop breeding. Notably, previous research has confirmed that the GST gene family plays a critical role not only in enhancing plant tolerance to salt stress but also in conferring resistance to a wide range of biotic and abiotic stresses, including heavy metal stress, drought stress, and pathogen stress [19,41]. For instance, the overexpression of the *Prosopis juliflora GSTU1* gene in tobacco enhanced the plant’s drought tolerance [42], while expressing the rice *OsGSTL2* gene in Arabidopsis increased resistance to heavy metals and other abiotic stresses [43]. These suggest that the GST gene family functions in a multifaceted role in plant resistance to abiotic stresses, making GST family genes promising targets for polyploid breeding aimed at efficiently combining multiple stress-resistance traits.

In conclusion, the transcriptomic data analysis of both salt-tolerant and salt-sensitive cotton varieties conducted in this study identified several potential salt stress-responsive genes within the GST gene family. It was confirmed that *GhGSTF9* likely serves as a positive regulatory factor in cotton’s tolerance to salt stress. These findings offer functional genes for the breeding of new salt-tolerant cotton varieties.

## Data Availability

The original contributions presented in the study are included in the article, further inquiries can be directed to the corresponding author/s.

## References

[1] van Zelm E., Zhang Y., Testerink C. (2020). Salt Tolerance Mechanisms of Plants. Annu. Rev. Plant Biol..

[2] Hu Y., Schmidhalter U. (2023). Opportunity and challenges of phenotyping plant salt tolerance. Trends Plant Sci..

[3] Mansour M.M.F., Hassan F.A.S. (2022). How salt stress-responsive proteins regulate plant adaptation to saline conditions. Plant Mol. Biol..

[4] Zhu J.K. (2001). Plant salt tolerance. Trends Plant Sci..

[5] Zhou H., Shi H., Yang Y., Feng X., Chen X., Xiao F., Lin H., Guo Y. (2024). Insights into plant salt stress signaling and tolerance. J. Genet. Genom..

[6] Liu X., Jiang W., Li Y., Nie H., Cui L., Li R., Tan L., Peng L., Li C., Luo J. (2023). FERONIA coordinates plant growth and salt tolerance via the phosphorylation of phyB. Nat. Plants.

[7] Wang C., Li X., Zhuang Y., Sun W., Cao H., Xu R., Kong F., Zhang D. (2024). A novel miR160a-GmARF16-GmMYC2 module determines soybean salt tolerance and adaptation. New Phytol..

[8] Ullah A., Sun H., Hakim, Yang X., Zhang X. (2018). A novel cotton WRKY gene, GhWRKY6-like, improves salt tolerance by activating the ABA signaling pathway and scavenging of reactive oxygen species. Physiol. Plant..

[9] Meng X., Liu S., Zhang C., He J., Ma D., Wang X., Dong T., Guo F., Cai J., Long T. (2023). The unique sweet potato NAC transcription factor IbNAC3 modulates combined salt and drought stresses. Plant Physiol..

[10] Wang F., Niu H., Xin D., Long Y., Wang G., Liu Z., Li G., Zhang F., Qi M., Ye Y. (2021). OsIAA18, an Aux/IAA Transcription Factor Gene, Is Involved in Salt and Drought Tolerance in Rice. Front. Plant Sci..

[11] Li Q., Xu F., Chen Z., Teng Z., Sun K., Li X., Yu J., Zhang G., Liang Y., Huang X. (2021). Synergistic interplay of ABA and BR signal in regulating plant growth and adaptation. Nat. Plants.

[12] Ma Z., Jin Y.M., Wu T., Hu L., Zhang Y., Jiang W., Du X. (2022). OsDREB2B, an AP2/ERF transcription factor, negatively regulates plant height by conferring GA metabolism in rice. Front. Plant Sci..

[13] Huang J., Ma S., Zhang K., Liu X., Hu L., Wang W., Zheng L. (2022). Genome-Wide Identification of Gramineae Brassinosteroid-Related Genes and Their Roles in Plant Architecture and Salt Stress Adaptation. Int. J. Mol. Sci..

[14] Kim J.H., Nguyen N.H., Jeong C.Y., Nguyen N.T., Hong S.W., Lee H. (2013). Loss of the R2R3 MYB, AtMyb73, causes hyper-induction of the SOS1 and SOS3 genes in response to high salinity in Arabidopsis. J. Plant Physiol..

[15] Zhao H., Yao P., Zhao J., Wu H., Wang S., Chen Y., Hu M., Wang T., Li C., Wu Q. (2022). A Novel R2R3-MYB Transcription Factor FtMYB22 Negatively Regulates Salt and Drought Stress through ABA-Dependent Pathway. Int. J. Mol. Sci..

[16] Rai G.K., Mishra S., Chouhan R., Mushtaq M., Chowdhary A.A., Rai P.K., Kumar R.R., Kumar P., Perez-Alfocea F., Colla G. (2023). Plant salinity stress, sensing, and its mitigation through WRKY. Front. Plant Sci..

[17] Yang Y., Guo Y. (2018). Unraveling salt stress signaling in plants. J. Integr. Plant Biol..

[18] Dixon D.P., Lapthorn A., Edwards R. (2002). Plant glutathione transferases. Genome Biol..

[19] Kumar S., Trivedi P.K. (2018). Glutathione S-Transferases: Role in Combating Abiotic Stresses Including Arsenic Detoxification in Plants. Front. Plant Sci..

[20] Horvath E., Bela K., Holinka B., Riyazuddin R., Galle A., Hajnal A., Hurton A., Feher A., Csiszar J. (2019). The Arabidopsis glutathione transferases, AtGSTF8 and AtGSTU19 are involved in the maintenance of root redox homeostasis affecting meristem size and salt stress sensitivity. Plant Sci..

[21] Jia B., Sun M., Sun X., Li R., Wang Z., Wu J., Wei Z., DuanMu H., Xiao J., Zhu Y. (2016). Overexpression of GsGSTU13 and SCMRP in Medicago sativa confers increased salt-alkaline tolerance and methionine content. Physiol. Plant..

[22] Dong Y., Li C., Zhang Y., He Q., Daud M.K., Chen J., Zhu S. (2016). Glutathione S-Transferase Gene Family in *Gossypium raimondii* and *G. arboreum*: Comparative Genomic Study and their Expression under Salt Stress. Front. Plant Sci..

[23] Sun Y., Tian Z., Zuo D., Wang Q., Song G. (2024). GhUBC10-2 mediates GhGSTU17 degradation to regulate salt tolerance in cotton (*Gossypium hirsutum*). Plant Cell Environ..

[24] Zhao H., Ge X., Li F. (2022). Revitalize China’s cotton industry. Nature.

[25] Dong Y., Hu G., Yu J., Thu S.W., Grover C.E., Zhu S., Wendel J.F. (2020). Salt-tolerance diversity in diploid and polyploid cotton (*Gossypium*) species. Plant J..

[26] Han M., Cui R., Wang D., Huang H., Rui C., Malik W.A., Wang J., Zhang H., Xu N., Liu X. (2023). Combined transcriptomic and metabolomic analyses elucidate key salt-responsive biomarkers to regulate salt tolerance in cotton. BMC Plant Biol..

[27] Liu Y., Wang L., Li X., Luo M. (2022). Detailed sphingolipid profile responded to salt stress in cotton root and the GhIPCS1 is involved in the regulation of plant salt tolerance. Plant Sci..

[28] Su Y., Liang W., Liu Z., Wang Y., Zhao Y., Ijaz B., Hua J. (2017). Overexpression of GhDof1 improved salt and cold tolerance and seed oil content in *Gossypium hirsutum*. J. Plant Physiol..

[29] Liang C., Meng Z., Meng Z., Malik W., Yan R., Lwin K.M., Lin F., Wang Y., Sun G., Zhou T. (2016). GhABF2, a bZIP transcription factor, confers drought and salinity tolerance in cotton (*Gossypium hirsutum* L.). Sci. Rep..

[30] Su Y., Guo A., Huang Y., Wang Y., Hua J. (2020). GhCIPK6a increases salt tolerance in transgenic upland cotton by involving in ROS scavenging and MAPK signaling pathways. BMC Plant Biol..

[31] Zhao G., Song Y., Wang Q., Yao D., Li D., Qin W., Ge X., Yang Z., Xu W., Su Z. (2020). *Gossypium hirsutum* Salt Tolerance Is Enhanced by Overexpression of *G. arboreum* JAZ1. Front. Bioeng. Biotechnol..

[32] Zhao H., Lin K., Ma L., Chen Q., Gan S., Li G. (2020). Arabidopsis NUCLEAR FACTOR Y A8 inhibits the juvenile-to-adult transition by activating transcription of MIR156s. J. Exp. Bot..

[33] Liang X., Li J., Yang Y., Jiang C., Guo Y. (2024). Designing salt stress-resilient crops: Current progress and future challenges. J. Integr. Plant Biol..

[34] Edwards R., Dixon D.P., Walbot V. (2000). Plant glutathione S-transferases: Enzymes with multiple functions in sickness and in health. Trends Plant Sci..

[35] Moons A. (2003). Osgstu3 and osgtu4, encoding tau class glutathione S-transferases, are heavy metal- and hypoxic stress-induced and differentially salt stress-responsive in rice roots. FEBS Lett..

[36] Xu J., Xing X.J., Tian Y.S., Peng R.H., Xue Y., Zhao W., Yao Q.H. (2015). Transgenic Arabidopsis Plants Expressing Tomato Glutathione S-Transferase Showed Enhanced Resistance to Salt and Drought Stress. PLoS ONE.

[37] Meng H., Zhao J., Yang Y., Diao K., Zheng G., Li T., Dai X., Li J. (2023). PeGSTU58, a Glutathione S-Transferase from *Populus euphratica*, Enhances Salt and Drought Stress Tolerance in Transgenic *Arabidopsis*. Int. J. Mol. Sci..

[38] Lv W., Jiang H., Cao Q., Ren H., Wang X., Wang Y. (2024). A tau class glutathione S-transferase in tea plant, CsGSTU45, facilitates tea plant susceptibility to *Colletotrichum camelliae* infection mediated by jasmonate signaling pathway. Plant J..

[39] Zhao H., Chen Y., Liu J., Wang Z., Li F., Ge X. (2023). Recent advances and future perspectives in early-maturing cotton research. New Phytol..

[40] Zhao H., Huang X., Yang Z., Li F., Ge X. (2023). Synergistic optimization of crops by combining early maturation with other agronomic traits. Trends Plant Sci..

[41] Nianiou-Obeidat I., Madesis P., Kissoudis C., Voulgari G., Chronopoulou E., Tsaftaris A., Labrou N.E. (2017). Plant glutathione transferase-mediated stress tolerance: Functions and biotechnological applications. Plant Cell Rep..

[42] George S., Venkataraman G., Parida A. (2010). A chloroplast-localized and auxin-induced glutathione S-transferase from phreatophyte *Prosopis juliflora* confer drought tolerance on tobacco. J. Plant Physiol..

[43] Kumar S., Asif M.H., Chakrabarty D., Tripathi R.D., Dubey R.S., Trivedi P.K. (2013). Expression of a rice Lambda class of glutathione S-transferase, OsGSTL2, in Arabidopsis provides tolerance to heavy metal and other abiotic stresses. J. Hazard. Mater..

